# Hsa_circ_0000479/Hsa-miR-149-5p/RIG-I, IL-6 Axis: A Potential Novel Pathway to Regulate Immune Response against COVID-19

**DOI:** 10.1155/2022/2762582

**Published:** 2022-08-30

**Authors:** Zahra Firoozi, Elham Mohammadisoleimani, Abbas Shahi, Mohammad Mehdi Naghizadeh, Ebrahim Mirzaei, Ali Ghanbari Asad, Zahra Salmanpour, Seyed Mohamad Javad Nouri, Yaser Mansoori

**Affiliations:** ^1^Department of Medical Genetics, Fasa University of Medical Sciences, Fasa, Iran; ^2^Department of Medical Genetics, Afzalipour School of Medicine, Kerman University of Medical Sciences, Kerman, Iran; ^3^Department of Medical Biotechnology, Fasa University of Medical Sciences, Fasa, Iran; ^4^Department of Immunology, School of Medicine, Tehran University of Medical Science, Tehran, Iran; ^5^Noncommunicable Diseases Research Center, Fasa University of Medical Sciences, Fasa, Iran; ^6^Department of Internal Medicine, Fasa University of Medical Sciences, Fasa, Iran

## Abstract

**Background:**

COVID-19, the disease caused by severe acute respiratory syndrome coronavirus 2 (SARS-CoV-2), has led to a global pandemic and mortality of people around the world. Some circular RNAs (circRNAs), one of the new types of noncoding RNAs (ncRNAs), act as competing endogenous RNAs (ceRNAs) and compete with mRNAs for shared miRNAs, to regulate gene expression. In the present study, we aimed to evaluate the expression and roles of hsa_circ_0000479/hsa-miR-149-5p/RIG-I, IL-6 in COVID-19 infection.

**Materials and Methods:**

After extraction of total RNA from peripheral blood mononuclear cells (PBMC) of 50 patients with symptomatic COVID-19, 50 patients with nonsymptomatic COVID-19, and 50 normal controls, cDNA synthesis was performed. Online *in silico* tools were applied to evaluate the interaction between the genes in the hsa_circ_0000479/hsa-miR-149-5p/RIG-I, IL-6 axis, and its role in COVID-19-related pathways. Quantification of the expression of these genes and confirmation of their interaction was done using the quantitative real-time PCR (qRT-PCR) technique.

**Results:**

The expression levels of hsa_circ_0000479, RIG-I, and IL-6 were increased in COVID-19 patients compared to healthy controls, while hsa-miR-149-5p expression was decreased. Moreover, there was a significant negative correlation between hsa-miR-149-5p and hsa_circ_0000479, RIG-I, IL-6 expressions, and also a positive expression correlation between hsa_circ_0000479 and IL-6, RIG-I. Then, bioinformatics tools revealed the role of hsa_circ_0000479/hsa-miR-149-5p/RIG-I, IL-6 axis in PI3K-AKT and STAT3 signaling pathways.

**Conclusion:**

Upregulation of hsa_circ_0000479, RIG-I, and IL-6, and downregulation of hsa-miR-149-5p, along with correlation studies, indicate that hsa_circ_0000479/hsa-miR-149-5p/RIG-I, IL-6 axis could play a role in regulating the immune response against SARS-CoV-2. However, more studies are needed in this area.

## 1. Introduction

One of the Beta-coronavirus genus, severe acute respiratory syndrome coronavirus 2 (SARS-CoV-2), which is highly transmittable, emerged in Wuhan, China, in December 2019, caused a pathogenic viral infection, called COVID-19. Due to its highly contagious nature, COVID-19 quickly became a global concern and was considered a serious threat to public health all over the world [1]. Fever, respiratory failure, cough, shortness of breath, and fatigue are the most common symptoms of COVID-19, although other symptoms such as gastrointestinal and neurological signs have been reported [2]. The SARS-CoV-2 genome, a single-stranded and positive-sense RNA virus, is about 29 Kb long and known to encode 30 different types of proteins [3]. The SARS-CoV-2 virus binds to the human angiotensin-converting enzyme 2 (ACE2) receptor through its spike protein and enters the cell, and some of the host cell proteases such as transmembrane serine protease 2 (TMPRSS2), enhance this process. Following, the SARS-CoV-2 genome replicates in the host cells' cytoplasm, which triggers inflammatory responses including cytokine storm [4]. Interleukin-6 (IL-6) is a cytokine produced from multiple cell types. When IL-6 binds to the membrane IL-6 receptor, it activates the JAK-STAT and PI3K-AKT signaling pathways, and thus plays a key role in causing cytokine storm in COVID-19 patients [5,6]. Also, the retinoic acid-inducible gene-I (RIG-I) signaling pathway is one of the major pathways that contribute to the production of type I/III Interferon (IFN). The IFN-I plays a key role in the antiviral immune response and promotes the expression of proinflammatory cytokines and it can lead to the cytokine storm in COVID-19 patients [7,8]. Thus, the exact regulation of proinflammatory and inflammatory cytokines seems essential during the host immune response against COVID-19 infection.

Also, it has been revealed that molecular mechanisms and dysregulation of various genes are responsible for the abnormal immune responses during COVID-19 infection [9,10]. Recently, it has been demonstrated that interactions of noncoding RNAs (ncRNAs), such as microRNAs (miRNAs), and circular RNAs (circRNAs), are associated with SARS-CoV-2 infection via the formation of circRNA/miRNA/mRNA axis [10].

The circRNAs are single-stranded closed-loop ribonucleic acids that are more stable than their linear structure. They have been implicated in multiple processes including miRNA sponging, modulating the transcription of target genes, and binding to RBP [11]. In this study, we mainly focus on the sponging properties of circRNAs for miRNAs. Increasing shreds of evidence indicate that circRNAs expressions are impaired in viral infections and proposed that circRNAs can be used as antiviral targets [11,12]. In the present study hsa_circ_0000479, also named circEPSTI1, was selected for further investigation. As previous studies have revealed that hsa_circ_0000479, also named circEPSTI1, is significantly overexpressed in systemic lupus erythematosus (SLE) and Hantaan virus (HTNV) infection, we decided to investigate the possible roles of hsa_circ_0000479 expression level in COVID-19 patients. [13,14].

The miRNAs, one of the small noncoding RNAs with 18–25 nucleotides in length, exert their role by blocking the mRNA translation or by degrading mRNA via binding to an open reading frame (ORF) or 3′- untranslated region (3′- UTR) [15]. Recent studies have shown that miRNAs could be a promising target for antiviral therapies [15,16].In this study, we picked out hsa-miR-149-5p, which strongly binds to the ACE2 [15], as a potential target of hsa_circ_0000479 [14,17].

Altogether, IL-6 and RIG-I were chosen because of their important roles in the immune response against SARS-CoV-2. In addition, with bioinformatics tools, the miRNA related to these genes involved in infectious diseases, especially COVID-19 infection, was assigned [18–20]. Although some studies revealed the regulatory mechanisms of competing for endogenous RNA (ceRNA) axis in SARS-CoV-2 infection, the expression pattern, and roles of hsa_circ_0000479/hsa-miR-149-5p/RIG-I, IL-6, which could affect several signaling pathways related to the immune system such as JAK-STAT and PI3K-AKT, are still unknown. Therefore, in this study, we aimed to evaluate the expression pattern and roles of hsa_circ_0000479/hsa-miR-149-5p/RIG-I, IL-6 ceRNA network in COVID-19 infection.

## 2. Materials and Methods

### 2.1. Patients and Sample Collection

We examined expression analysis in three groups of negative controls, individuals with severe-symptomatic and nonsymptom COVID-19, and each group contained 50 samples. Confirmation of these groups was performed by an expert physician based on symptoms and qRT-PCR results. Patients included those who were referred to or hospitalized in Valiasr and Shariati hospitals, Fasa, Fars, Iran. The negative control group consists of individuals who had no infectious or immune-related diseases, such as autoimmunity, allergy, cancer, and liver diseases. Blood samples were collected in EDTA tubes and transferred to the laboratory for RNA extraction in less than three hours. All the participants have signed a consent form. This study was approved by the ethics committee of Fasa University of Medical Sciences (ethical code: IR.FUMS.REC.1399.215). We have followed all ethical approvals for this study. Signed informed consent form was collected from all patients.

### 2.2. RNA Extraction and cDNA Synthesis

Total RNA was extracted from the human peripheral blood mononuclear cells (PBMC) samples using the TriSol isolation reagent (Invitrogen, Thermo Fisher) according to the manufacturer's instructions. The RNA integrity and quantity were assessed via gel electrophoresis and spectrophotometry (NanoDrop 2000, Thermo Scientific, USA), respectively. Total RNA was reverse transcribed to first-strand cDNA using the PrimeScript™ RT Reagent Kit (BioFact™, Cat.No: BR441-096) according to the manufacturer's protocol.

### 2.3. Quantitative Real-Time PCR

Quantitative real-time PCR was carried out using RealQ Plus 2x Master Mix Green with High ROX (Amplicon, Cat.No: A325402-25). The reaction volume was 15 *μ*l including 7.5 *μ*l master mix, 1 *μ*l of cDNA, 0.75 *μ*l of each primer, and 5 *μ*l DNase-free dH2O. All reactions were performed according to 45 cycles of 95°C for 20 seconds, and then 60°C for 30 seconds. Internal control for quantitative applications of miRNA was U48 and endogenous control for circRNA and mRNA was ACTB. Hsa_circ_0000479, hsa-miR-149-5p, IL-6, RIG-I, ACTB, and U48 primer sequences were shown in Table 1. Gene expression was quantitated using the 2^−∆∆CT^ method.

### 2.4. Statistical Analysis

SPSS Statistics software v.26 was used for data analysis and GraphPad Prism v.8 was applied for graphical visualization. The nonparametric Kruskal Wallis H test was employed to compare the expression level among the three different groups of samples containing symptomatic COVID-19, nonsymptomatic COVID-19, and healthy control. Comparisons between these groups in terms of sex and blood group were performed using the Chi-square test and in terms of age using the one-way ANOVA test. The correlation between the hsa_circ_0000479/hsa-miR-149-5p/RIG-I, IL-6 pathway genes was assessed by the Spearman correlation coefficient test. The statistically significant level was considered *P* value less than 0.05.

### 2.5. In Silico Predictions

In this study we evaluated the hsa_circ_0000479/hsa-miR-149-5p/RIG 1, IL-6 axis expression. First, the interaction between circRNA and miRNA was assessed by the Circular RNA Interactome (https://circinteractome.nia.nih.gov/). Second, we used miRTargetLink (https://ccb-web.cs.uni-saarland.de/mirtargetlink/), miRTarBase (miRTarBase: the experimentally validated microRNA-target interactions database (cuhk.edu.cn)), and miRWalk2.0 (http://zmf.umm.uni-heidelberg.de/apps/zmf/mirwalk2/) to investigate mRNA-miRNA interaction. Third, gene ontology (GO) enrichment analysis of the ceRNA network was performed by miRPathDB 2.0 (https://mpd.bioinf.uni-sb.de/) and KEGG (https://www.genome.jp/kegg/).

## 3. Results

### 3.1. Patient's Basic and Demographic Data

Fifty symptomatic COVID-19 included 20 (40%) females and 30 (60%) males, with a mean age of 41.46 years old, and 50 nonsymptomatic COVID-19 comprise of 23 (46%) females and 27 (54%) males, with a mean age of 48.84 years old participated in this study. Also, there were 27 (54%) males and 23 (46%) females in the healthy control group with a mean age of 43.16. There was a match between patients and the control group in terms of age, sex, and blood group type (Table 2). There were 10 (20%) and 12 (24%) cases with cardiovascular disease and 5 (10%) and 2 (4%) subjects with immunodeficiency in the group of patients with symptomatic and nonsymptomatic COVID-19, respectively, (Table 2). The different symptoms of patients with symptomatic COVID-19 are summarized in Table 3.

### 3.2. Comparison of the Expression Level of hsa_circ_0000479, Hsa-miR-149-5p, RIG-I, And IL-6 in the Two Different Subgroups of COVID-19 Patients (Symptomatic, Nonsymptomatic) and Healthy Controls

**Figure 1:**
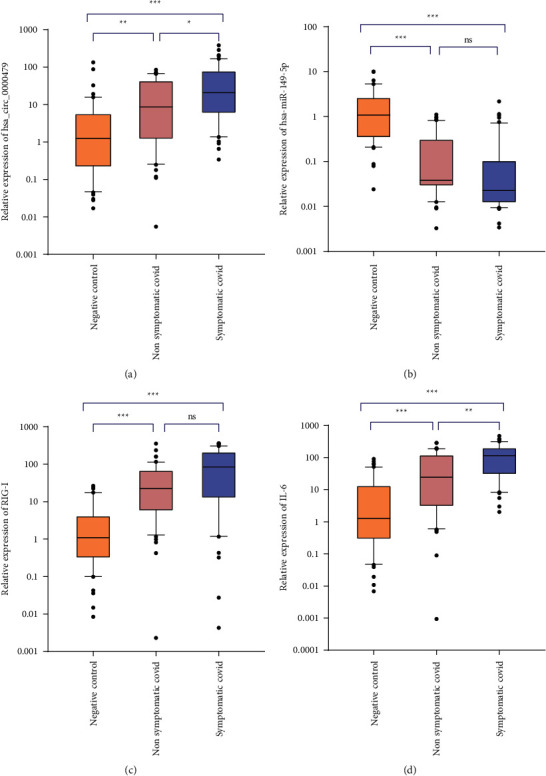
The box and whisker plot (10–90 percentile) of the relative expression levels of hsa_circ_0000479, hsa-miR-149-5p, IL-6, and RIG-I (ns: *P* > 0.05^*∗*^*P* ≤ 0.05; ^*∗∗*^*P* ≤ 0.01; ^*∗∗∗*^*P* < 0.001). (a) Upregulation of hsa_circ_0000479 in COVID-19 patients compared to healthy. (b) Down expression of hsa-miR-149-5p in COVID-19 patients compared to healthy controls. (c) Upregulation of RIG-I in COVID-19 patients compared to healthy controls. (d) Upregulation of IL-6 in COVID-19 patients compared to healthy controls.

By using real-time PCR, the expression level of selected genes was evaluated in COVID-19 patients and healthy control samples. The expression of hsa_circ_0000479 was significantly upregulated in symptomatic COVID-19 samples in comparison to other groups, and also it was upregulated in nonsymptomatic patients compared to negative controls (Table 4, Figure 1(a)). The evaluation of hsa-miR-149-5p expression showed significant downregulation in symptomatic COVID-19 patients compared to negative controls and also the expression of hsa-miR-149-5p was significantly downregulated in nonsymptomatic COVID-19 patients compared to negative controls (Table 4, Figure 1(b)). The expression level of RIG-I and IL-6 were clearly higher in patients with symptomatic COVID-19 compared to healthy controls, and also, they were upregulated in nonsymptomatic patients in comparison to the control group. Moreover, IL-6 expression was significantly higher in symptomatic in comparison to nonsymptomatic COVID-19 patients. However, RIG-I did not show a significant expression in this context (Table 4, Figure 1(c), 1(d)).

### 3.3. Correlation Analysis between hsa_circ_0000479 and Hsa-miR-149-5p

**Figure 2:**
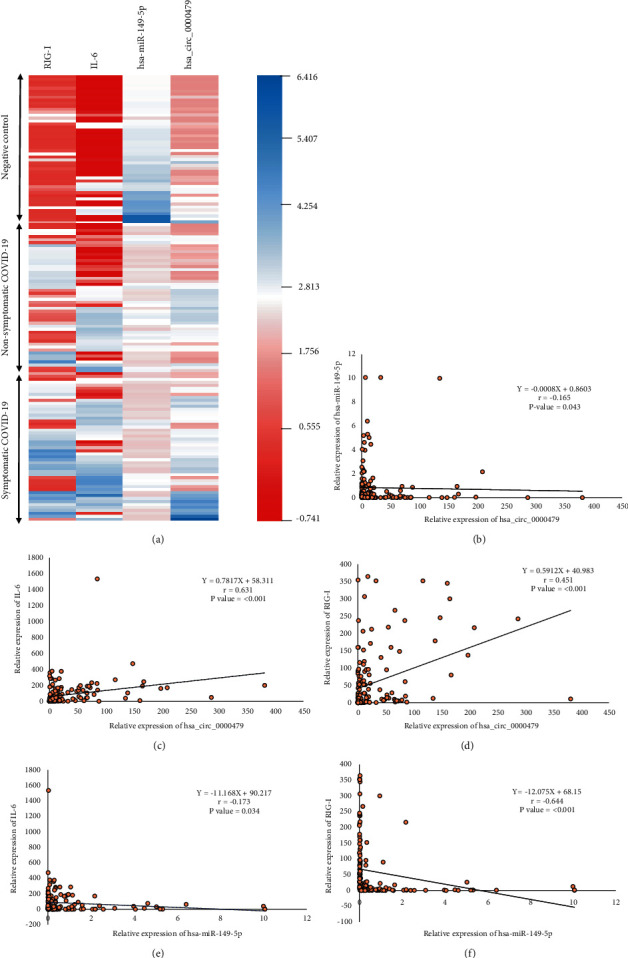
(a) The heatmap analysis. (b) The negative correlation between hsa_circ_0000479 and hsa-miR-149-5p expression levels in COVID-19 (*r* = −0.165, *P* value = 0.043). (c) The positive correlation between hsa_circ_0000479 and IL-6 expression levels in COVID-19 (*r* = 0.631, *P* value = ˂0.001). (d) The positive correlation between hsa_circ_0000479 and RIG-I expression levels in COVID-19 (*r* = 0.451, *P* value=˂0.001). (e) The negative correlation between hsa-miR-149-5p and IL-6 expression levels in COVID-19 (*r* = −0.173, *P* value = 0.034). (f) The negative correlation between hsa-miR-149-5p and RIG-I expression levels in COVID-19 (*r* = −0.644, *P* value = ˂0.001).

**Figure 3:**
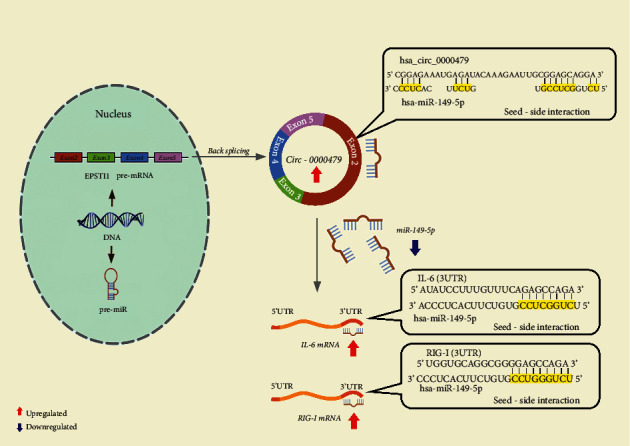
The schematic diagram demonstrates that hsa_circ_0000479 could modulate the expression of IL-6 and RIG-I through sponging of hsa-miR-149-5p in SARS-CoV-2 infection.

We conducted the correlation analysis between hsa_circ_0000479 and hsa-miR-149-5p, to decide whether hsa_circ_0000479 may act as a sponge for hsa-miR-149-5p or not. A negative correlation between the expression of these ncRNAs was observed (*r* = −0.165, *P* value = 0.043, Figure 2(b)). It was bioinformatically predicted that IL-6 and RIG-I could be considered as hsa_circ_0000479/hsa-miR-149-5p targets. The results of correlation analysis showed a positive correlation between hsa_circ_0000479 and IL-6, RIG-I (*r* = 0.631, *P* value = <0.001/*r* = 0.451, *P* value = <0.001, Figures 2(c), 2(d)) as well as the negative expression correlation between hsa-miR-149-5p and IL-6, RIG-I (*r* = -0.173, *P* value = 0.034/*r* = −0.644, *P* value = <0.001, Figures 2(e), (f)). Furthermore, the heatmap analysis derived from the expression pattern of hsa_circ_0000479/hsa-miR-149-5p/IL-6, RIG-I axis reveals a distinctive expression profile between COVID-19 samples and negative controls (Figure 2(a)). Altogether, our results are indicative of the importance of the hsa_circ_0000479/hsa-miR-149-5p/IL-6, RIG-I pathway in COVID-19 (Figure 3).

### 3.4. Bioinformatics Prediction of Pathways Involved in hsa_circ_000479/Hsa-miR-149-5p/RIG1, IL6 Axis

**Figure 4:**
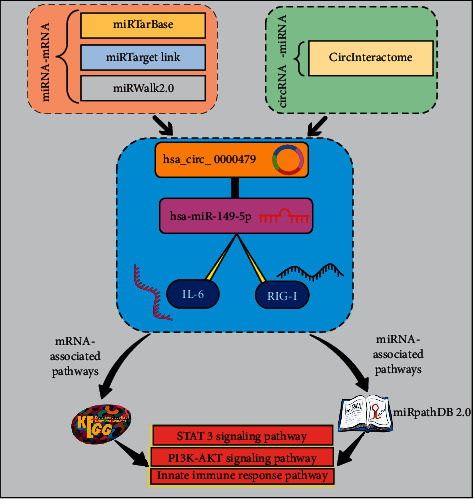
A schematic representation of a workflow for bioinformatics analysis.

According to in silico results, the hsa_circ_0000479/hsa-miR-149-5p/RIG-I, IL-6 triple network has been generated using circRNA-miRNA pair and miRNA-mRNA pair. Additionally, to understand the function of the ceRNA network, mRNA and miRNA-associated pathways were investigated. We used the miRPathDB and KEGG database to detect the pathways that are related to hsa-miR-149-5p and mRNAs (IL-6 and RIG-I) in ceRNA, respectively. In addition, significant pathways related to them were innate immune response, PI3K-AKT, and STAT3 signaling pathways (Figure 4).

## 4. Discussion

SARS-CoV-2 is one of the most transmittable pathogens belonging to the family of Coronaviridae (CoVs), which has developed into a pandemic and is associated with high rates of mortality and morbidity.

Despite rapid progression and the emergence of new mutations in its genome around the world, our knowledge about virulence factors of SARS-CoV-2 and its specific therapeutic strategies is still limited [1,21]. Therefore, augmenting our knowledge of molecular etiology is vital to finding innovative approaches for the control and treatment of this disease.

In recent years, a novel type of noncoding RNAs known as circRNAs has attracted incredible attention owing to their high stability, cell-specific expression, conservation across species, and great abundance [22]. The increasing amount of evidence suggests the important role of circRNAs in viral infections. One of the pivotal roles of circRNAs in viral infections is related to their regulatory effect on innate immune response-related miRNAs [11]. Additionally, circRNAs can regulate the innate immune response by reacting with dsRNA binding antiviral proteins, which are important substances in the host innate immune system. Retinoic acid-inducible gene-I (RIG-I) is a cytosolic RNA-sensing protein that recognizes viral dsRNA and stimulates the innate immune system and inhibits viral infection [23, 24]. Assessing the molecular pathways by which circRNAs affect viral infection can enable a deeper understanding of the etiology of infectious diseases like COVID-19 and provide early diagnosis and treatment strategies [11]. Among the circRNAs that had been shown to play a significant role in infectious diseases [11, 25], we picked out hsa_circ_0000479 for further examination.

The hsa_circ_0000479 is a circular transcript derived from the epithelial‐stromal interaction 1 (EPSTI1) gene with 375 bp length [26]. In upper airway samples and blood leukocytes of SARS‐CoV‐2‐infected patients, it has been reported that EPSTI1 is significantly overexpressed compared to patients without SARS‐CoV‐2 infection, and could have a helpful effect on antiviral therapy in COVID-19 [27]. Another study has shown that EPSTI1 acted as a regulator of the inflammatory response in a mouse model by activating macrophages and subsequently adjusting the cStat1 and p65 pathways [28]. In a study by Shaath et al. the expression levels of EPSTI1 were significantly higher in neutrophils and inflammatory macrophages, which suggests the role of this gene in the antiSARS‐CoV‐2 inflammatory response [29]. However, the expression and function of circRNAs derived from the EPSTI1 gene in COVID-19 are still chiefly unknown. Our experimental results indicate that hsa_circ_0000479 is significantly upregulated in symptomatic COVID-19 patients compared to other groups including nonsymptomatic COVID-19 patients and negative controls. There are few studies about the functions of this circRNA in diseases in which the immune and inflammatory responses are involved. In a study, Qing et al. evaluated the expression profile of circRNAs in systemic lupus erythematosus (SLE). First, by using microarray analysis, they found 11 differentially expressed circRNAs in SLE, including hsa_circ_0000479. In the second stage, they used qRT-PCR technique to assess these circRNAs expressions in PBMC of 23 SLE and 23 healthy controls, and their results showed that the expression of hsa_circ_0000479 was significantly increased in patients with SLE, and also it is generally stable in PBMC. In addition, the expression level of hsa_circ_0000479 is inversely correlated with the level of complement 3 (C3) and can generally be used as a diagnostic and prognostic marker in SLE [13]. In another study, Shuang et al. performed RNA sequencing to evaluate the circRNA/miRNA/mRNA pathways in HTNV-infected (Hantaan virus) and mock-infected human umbilical vein endothelial cells (HUVECs). Next, the qRT-PCR technique was used for data validation. The qRT-PCR results were significantly in agreement with RNA sequencing data, which in the case of hsa_circ_0000479 showed an increase in expression in HTNV-infected cells. They also found that hsa_circ_0000479 indirectly regulates RIG-I expression by sponging hsa-miR-149-5p, thereby affecting the activity of the host immune system and suppressing Hantaan virus replication. Similar to ours, their results show the upregulation of hsa_circ_0000479 and RIG-I, and downregulation of hsa-miR-149-5p [14]. These events suggest that hsa_circ_0000479 could have potential functions in regulating the immune system's response against viruses. Nevertheless, little evidence is available about the roles of this circRNA in COVID-19 and more studies are needed to elucidate its exact roles.

Recently, copious studies have demonstrated that circRNAs can participate in ceRNA networks by sponging miRNAs, and indirectly regulating mRNAs expression [30]. Therefore, we applied online bioinformatics tools to find potential targets for hsa_circ_0000479. Among the important and functional miRNAs which play a role in the immune responses against viral infections, hsa-miR-149-5p was selected for further analysis. Our qRT-PCR results indicated the downregulation of hsa-miR-149-5p in symptomatic COVID-19 patients compared to nonsymptomatic COVID-19 patients and negative controls. Moreover, by using bioinformatics tools, we found that IL-6 and RIG-I are potential targets for hsa-miR-149-5p as a key player in the immune response against COVID-19. The expression analysis of these two mRNAs revealed that IL-6 and RIG-I have significantly higher expressions in COVID-19 patients. The conserved 3′-UTR sequence of IL-6 and RIG-I for hsa-miR-149-5p and conserved sequence of hsa_circ_0000479 for hsa-miR-149-5p is exhibited in Figure 3. Kyoto Encyclopedia of Genes and Genomes (KEGG) enrichment analysis demonstrates that RIG-I and IL-6 are principally involved in the innate immune response, PI3K-AKT, and STAT3 signaling pathways in COVID-19. Moreover, bioinformatics tools revealed that hsa-miR-149-5p is also engaged in these pathways. Altogether, it seems that the miR-149-5p/IL-6, RIG-I can act as one of the most eminent interactions in the regulation of PI3K-AKT and STAT3 signaling pathways, which are important in COVID-19 infection.

In the following step, we evaluated the expression correlation of these genes. Our findings show a significant negative correlation between the expression of hsa-miR-149-5p and hsa_circ_0000479, IL-6, RIG-I, and also a positive correlation between the expression levels of hsa_circ_0000479 and IL-6, RIG-I. These data suggest a novel potential molecular relationship between the hsa_circ_0000479, miR-149-5p, IL-6, and RIG-I, as a ceRNA regulatory network in COVID-19.

A recent study has predicted that hsa-miR-149-5p has strong potential binding sites against angiotensin-converting enzyme 2 (ACE2), which mediates SARS-CoV-2 cellular entry, and could regulate the expression of ACE2 by direct binding or through some other pathways, and also can be used as a marker for therapeutic purposes in SARS-CoV-2 infection [31]. Another study predicted multiple miRNAs which could bind the SARS-CoV-2 RNA genome with high affinity, including hsa-miR-149-5p [32].

Interleukin-6 (IL-6) is one of the most important cytokines which is secreted by several types of immune cells such as T cells, macrophages, endothelial cells, fibroblasts, and monocytes, and its production is associated with various inflammatory diseases and is also engaged in inflammatory response against SARS-CoV-2, known as the cytokine storm [33,34]. In addition, it was reported that severe COVID-19 patients have higher levels of IL-6 and its expression level is related to pulmonary inflammation and extensive lung damage [35]. The RIG-I plays an important role in the activation of the downstream signaling pathways, causing the production of interferons (IFN), and resulting in the restriction of SARS-CoV-2 replication in human lung cells [36].

Collectively, we suggest that up-expressed hsa_circ_0000479 could modulate the expression of IL-6 and RIG-I through sponging of hsa-miR-149-5p in COVID-19. As IL-6 and RIG-I are vital genes in the immune response in COVID-19 infection, regulation of these genes' expression could affect the severity of symptoms. Hence, the hsa_circ_0000479/hsa-miR-149-5p/IL-6, RIG-I axis might be an encouraging therapeutic target for COVID-19 patients.

## 5. Conclusion

In conclusion, by combining the experimental analysis and using bioinformatics tools, we constructed a circRNA/miRNA/mRNA network involved in PI3K-AKT and STAT3 signaling pathways in COVID-19. The significant upregulation of IL-6 and RIG-I in symptomatic COVID-19 patients could be mediated by hsa_circ_0000479 through sponging the hsa-miR-149-5p. To the best of our knowledge, this is the first study that develops a new perspective on the roles of this ceRNA axis in regulating the immune system response against SARS-CoV-2. Nevertheless, further studies are required to explore the clinical applications of these findings.

## Figures and Tables

**Table 1:** qRT-PCR primers sequences.

Gene symbol	Primer sequence (5′–3′)
**hsa_circ_0000479 (F)**	GAGCATCAGCAATACACAAGTG
**hsa_circ_0000479 (R)**	TTTAGCTCTGTTCTGCTCCTTC
**hsa-miR-149-5p (F)**	GTCTGGCTCCGTGTCTTC
**hsa-miR-149-5p (RT)**	GTCGTATCCAGTGCAGGGTCCGAGGTA
	TTCGCACTGGATACGACGGGAGT
**hsa-miR-149-5p and U48 (R)**	GTGCAGGGTCCGAGGT
**U48 (F)**	AGGTAACTCTTGAGTGTGTCGCT
**U48 (RT)**	GTCGTATCCAGTGCAGGGTCCGAGGT
	ATTCGCACTGGATACGACGGTCAGA
**IL-6 (F)**	AGACAGCCACTCACCTCTTCAG
**IL-6 (R)**	TTCTGCCAGTGCCTCTTTGCTG
**RIG-I (F)**	ATGGGACGAAGCAGTATTTAG
**RIG-I (R)**	GCTTGGGATGTGGTCTACTC
**ACTB (F)**	TGGAACGGTGAAGGTGACAG
**ACTB (R)**	CTGTAACAACGCATCTCATATTTGG

**Table 2:** Baseline and demographic characteristics of participants **(***P*values 1: in comparison with negative control, 2: in comparison with nonsymptomatic COVID-19).

		Negative control Count	Nonsymptomatic COVID-19			Symptomatic COVID-19		
			N %	Count	N %	Count	N %	*P*-value
Sex	Male	27	54.0%	27	54.0%	30	60.0%	0.784
	Female	23	46.0%	23	46.0%	20	40.0%	

Blood group	A+	13	26.0%	13	26.0%	12	24.0%	0.319
	A−	6	12.0%	2	4.0%	4	8.0%	
	B+	11	22.0%	18	36.0%	11	22.0%	
	B−	0	0.0%	5	10.0%	3	6.0%	
	O+	13	26.0%	8	16.0%	13	26.0%	
	O−	4	8.0%	4	8.0%	4	8.0%	
	AB+	3	6.0%	0	0.0%	3	6.0%	

Underlying disease	None	0	0.0%	36	72.0%	35	70.0%	0.477
	Cardiovascular disease	0	0.0%	12	24.0%	10	20.0%	
	Immune deficiency	0	0.0%	2	4.0%	5	10.0%	

*Age*	*Mean*	*SD*	*P-value1*	*P-value2*

Negative control	43.16	15.61						
Nonsymptomatic COVID-19	48.84	18.06	0.192					
Symptomatic COVID-19	41.46	13.85	0.854	0.064		

**Table 3:** Different signs and features of symptomatic COVID-19 patients.

Symptomatic COVID-19 patients			
		Count	Column N (%)
Fever-tremor	Yes	26	52.0
Cough	Yes	33	66.0
Shortness breath	Yes	28	56.0
Weakness	Yes	34	68.0
Pain bruising	Yes	32	64.0
Dizziness	Yes	13	26.0
Sore throat	Yes	27	54.0
Runny noise	Yes	14	28.0
Diarrhea	Yes	12	24.0
Vomiting-nausea	Yes	10	20.0
Headache	Yes	27	54.0
Chest pain	Yes	25	50.0
Stomachache	Yes	21	42.0
Joint pain	Yes	23	46.0
Red conjunctiva	Yes	3	6.0
Fatigue	Yes	22	44.0
BMI	≤25	25	50.0
	25–29	17	34.0
	≥30	8	16.0

**Table 4:** The expression levels of hsa_circ_0000479, hsa-miR-149-5p, IL-6, and RIG-I in negative controls, nonsymptomatic COVID-19, and symptomatic COVID-19 patients **(***P*values 1: in comparison with negative control, 2: in comparison with nonsymptomatic COVID-19).

Gene name	Group	N	Mean	SD	Median	*P*-value1	*P*-value2
**hsa_circ_0000479**	Negative control	50	8.073	22.661	1.238		
	Nonsymptomatic COVID-19	50	19.903	25.199	8.681	0.003	
	Symptomatic COVID-19	50	58.625	81.253	20.608	<0.001	0.012

**hsa-miR-149-5p**	Negative control	50	2.123	2.600	1.088		
	Nonsymptomatic COVID-19	50	0.207	0.296	0.038	<0.001	
	Symptomatic COVID-19	50	0.179	0.395	0.023	<0.001	0.163

**RIG-I**	Negative control	50	4.463	7.289	1.091		
	Nonsymptomatic COVID-19	50	48.413	66.450	22.013	<0.001	
	Symptomatic COVID-19	50	121.275	113.371	88.392	<0.001	0.090

**IL-6**	Negative control	50	12.744	22.678	1.266		
	Nonsymptomatic COVID-19	50	65.659	78.441	24.345	<0.001	
	Symptomatic COVID-19	50	136.516	117.751	120.641	<0.001	0.005

## Data Availability

The data used to support the findings of this study can be obtained from the corresponding author upon request.

## Abbreviations

qRT-PCR: Quantitative real-time PCR
SARS-CoV-2: Severe acute respiratory syndrome coronavirus 2
CircRNAs: Circular RNAs
ncRNAs: Noncoding RNAs
ceRNAs: Competing endogenous RNAs
miRNAs: MicroRNAs
PBMC: Peripheral blood mononuclear cells
ACE2: Angiotensin-converting enzyme 2
TMPRSS2: Transmembrane serine protease 2
IL-6: Interleukin-6
RIG-I: Retinoic acid-inducible gene-I
IFN: Interferon
3′- UTR: 3′: untranslated region
SLE: Systemic lupus erythematosus
HTNV: Hantaan virus
ORF: Open reading frame
GO: Gene ontology
EPSTI: Epithelial‐stromal interaction 1
C3: Complement 3
HUVECs: Human umbilical vein endothelial cells
KEGG: Kyoto Encyclopedia of Genes and Genomes.
